# Kinetic Study of the Oxidative Thermal Degradation of Polymer Composites Loaded with Hybrid Nanostructured Forms of Carbon: Correlation with Electrical and Morphological Properties

**DOI:** 10.3390/polym18101150

**Published:** 2026-05-08

**Authors:** Annalisa Paolone, Francesco Trequattrini, Marialuigia Raimondo, Liberata Guadagno, Stefano Vecchio Ciprioti

**Affiliations:** 1 Consiglio Nazionale delle Ricerche—Istituto dei Sistemi Complessi, U.O.S. La Sapienza, Piazzale A. Moro 5, 00185 Roma, Italy; annalisa.paolone@cnr.it (A.P.); francesco.trequattrini@uniroma1.it (F.T.); 2 Dipartimento di Fisica, Sapienza Università di Roma, Piazzale A. Moro 5, 00185 Roma, Italy; 3 Dipartimento di Ingegneria Industriale, Università di Salerno, Via Giovanni Paolo II, 132, 84084 Fisciano, Italy; lguadagno@unisa.it; 4 Dipartimento di Scienze di Base ed Applicate per l’Ingegneria, Sapienza Università di Roma, Palazzina RM017, Via del Castro Laurenziano 7, 00161 Roma, Italy

**Keywords:** hybrid nanostructures, carbon nanotubes, graphene filler, oxidative thermal degradation, isoconversional kinetic methods

## Abstract

The present research article deals with the thermal degradation study of epoxy resins filled with hybrid nanostructured forms of carbon under oxidative conditions. In particular, the formulated polymer composites (denoted as HYB_0.1%_CNTs:GNs and HYB_0.5%_CNTs:GNs, respectively) consist of two kinds of fillers, namely multi-walled carbon nanotubes (CNTs) and graphene nanosheets (GNs), mixed together with two different total mass amounts: 0.1 and 0.5%. In both kinds of nanocomposites, three different CNT:GN mixing ratios were considered (5:1, 1:1, and 1:5, respectively), thus providing a total of six hybrid samples. The thermal behavior of these samples was studied by simultaneous thermogravimetry and differential thermal analysis (TG/DTA) under flowing air, and two processes took place in distinct temperature ranges. In each step, about 50% of mass loss is detected with an exothermic effect in the corresponding DTA curve, with the second one accompanied by an intense heat release. The kinetic analysis of the two-stage oxidative thermal degradation was investigated using a model-free isoconversional approach. A non-Arrhenian behavior of the temperature function k(T) was assumed, and lifetime prediction was estimated at temperatures close to those of the possible applications. Isoconversional analysis shows nearly constant activation energies for all composites except HYB_0.1%_5:1 (from 142 to 96 kJ·mol^−1^), while lifetime predictions indicate that thermal stability increases with graphene content at 0.1% loading (HYB_0.1%_1:5) and with CNT content at 0.5% loading (HYB_0.5%_5:1), with uncertainties below 7%. Finally, because of the π–π bond interactions between the CNTs and the GNs dispersed in the epoxy resin matrix, an effective and remarkable electrical performance was found and a correlation with both electrical and morphological properties was established. In this regard, Tunneling Atomic Force Microscopy (TUNA) proved to be particularly powerful in allowing the simultaneous mapping of topography and localized conductive networks with exceptional sensitivity to nanofiller dispersion, such as CNTs and GNs. DC conductivity increased by up to nine orders of magnitude at 0.1 wt% hybrid loading (up to 3.73 × 10^−4^ S/m vs. 1.06 × 10^−13^ S/m for CNT-only), with nanoscale TUNA currents (−1.9 to 4.5 pA) mirroring macroscopic trends, while at 0.5 wt% all hybrids reached 10^−2^ S/m, indicating reduced synergy once a fully developed conductive network is established.

## 1. Introduction

Hybrid nanostructured carbon fillers (like graphene, carbon nanotubes, and carbon black) strongly influence both the electrical conductivity and morphology of polymer composites. Their synergistic interactions create percolated conductive networks while also modifying microstructure, leading to improved mechanical, triboelectric, and sensing properties [1].

Combining carbon nanotubes (CNTs) and graphene nanoparticles (GNPs) creates a synergistic effect that significantly lowers the percolation threshold in polymer composites, forming more efficient 3D conductive networks through CNTs bridging the 2D GNP sheets, allowing for high conductivity at much lower filler loadings than either filler alone, especially when their aspect ratios and content ratios are optimized [2,3,4].

Before undergoing curing, the presence of epoxy functional groups bound to the aliphatic, aromatic, or heterocyclic backbone of epoxy resins caused an improvement of the mechanical strength and thermal stability of these materials. For these reasons, they are commonly used in aeronautical, aerospace, and other engineering fields as fire-retardant additives [5,6].

An exhaustive kinetic analysis of the oxidative thermal degradation process occurring in epoxy resins-based composites made of hybrid nanostructured forms of carbon aims at selecting the most appropriate synthesis procedure or to fine-tune the desired properties to improve the performance for defined applications [7,8,9,10].

This study is important, as it explores the critical relationship between thermal stability and electrical properties of epoxy-based composites enhanced with hybrid fillers, specifically carbon nanotubes and graphene nanoparticles. The main focus is to investigate how varying the amounts and ratios of these nanofillers affects the kinetics of oxidative degradation, thereby improving the material’s performance for applications in demanding environments. By establishing this correlation, the research aims to contribute valuable insights into the design of advanced materials with superior mechanical and thermal characteristics, essential for industries such as aerospace, electronics, and automotive.

Thus, one of the main aims of this study is to correlate the results of this kinetic investigation on the oxidative degradation with the relative amount of the two fillers (CNTs and GNs, respectively) in the epoxy resins-based composites having two different total mixed filler amounts (0.1 and 0.5 wt%). It is hypothesized that varying the ratios of carbon nanotubes (CNTs) to graphene nanosheets (GNs) in epoxy composites will significantly influence both the electrical conductivity and oxidative thermal stability of the materials. Specifically, an optimal CNT/GN ratio will be identified that maximizes the formation of a continuous conductive network, thereby enhancing electrical performance while simultaneously improving thermal degradation kinetics through effective heat dissipation and oxygen barrier properties. This hypothesis is grounded in the understanding that the synergistic effects of CNTs and GNs can lead to improved material properties due to their distinct structural and electrical characteristics, which interact to provide enhanced stability and conductivity.

This clear statement would guide the research direction and provide a basis for the expected outcomes, allowing for a more focused investigation into the effects of varying CNT/GN ratios on the performance of the epoxy composites.

In this work, we focused on the reaction time (tα) for the second step of oxidative decomposition at low conversion, relating it to factors such as activation energy (*E_a_*), morphology, or filler content. As filler content increases, tα at a given conversion (α) increases, suggesting greater stability. This is attributable to improved dispersion, cross-linking, and conductivity. For low *α*, *tα* tends to correlate with filler content: a higher *tα* indicates delayed degradation. Subsequently, we speculated on a correlation with morphology and electrical properties. From a literature search, the estimated reaction time *tα* values for the second step of oxidative decomposition at low conversions systematically increase with the content of CNT/graphene hybrid fillers [11,12,13]. This behavior indicates a slowdown in oxidation kinetics in the initial phase of the main decomposition, reflecting the protective role of the hybrid carbon network [14,15,16].

The longer *tα* values are consistent with the improved dispersion and interconnected morphology observed by Scanning Electron Microscopy (SEM) morphological analysis and, in our case, for the first time with TUNA, and with the higher electrical conductivity, both indicators of the formation of an efficient percolated carbon structure that acts as an oxygen diffusion barrier and a stabilizing scaffold for the coal.

By analyzing *tα* at our filler loadings (0.1 and 0.5 wt% of the CNT/GN mixture with different combination ratios), we can evaluate how the oxidative degradation kinetics evolve as a function of nanoscale electrical properties. In the second oxidative step, *tα* represents the time required to reach a given conversion α during the main oxidation and char-consumption stage of the epoxy–carbon network. At low degrees of conversion, *tα* reflects how rapidly the oxidative attack begins to progress once this step is initiated. Higher *tα* values indicate a slower oxidation rate and therefore greater resistance to oxidative degradation, whereas lower tα values correspond to faster oxidation and a less protective network [17].

In epoxy nanocomposites containing CNT/GN hybrid fillers, effective dispersion and network formation typically lead to increased tα at low α compared to the neat resin [18,19,20]. This behavior suggests a delayed onset of oxidation due to the barrier effect created by the tortuous diffusion path and the stabilized carbonaceous char, which together hinder oxygen ingress and volatile release. Conversely, when dispersion is poor or the loading is sub-optimal, *tα* may remain unchanged or even decrease relative to the neat epoxy, indicating that agglomerates or disconnected networks fail to provide an effective barrier and may promote localized degradation.

Regarding the kinetic study of oxidative thermal degradation, when polymer composites are loaded with hybrid carbon nanostructures (such as mixtures of carbon nanotubes (CNTs) and graphene nanosheets (GNs), their thermal degradation kinetics change in predictable ways, primarily driven by a synergistic effect that increases the thermal stability of the polymer matrix [21,22,23,24]. The changes in thermal degradation kinetics typically follow these patterns:

- Increased activation energy (*Ea*): the addition of hybrid nanofillers acts as “restriction sites” that reduce the segmental mobility of polymer chains, thereby raising the activation energy required for degradation to initiate [25].
- Shift to Higher Degradation Temperatures: the presence of both 1D CNTs and 2D GNs creates a 3D conductive network, which improves thermal conductivity and allows for better heat dissipation, shifting the maximum degradation rate to higher temperatures [26].
- Enhanced Char Formation (Barrier Effect): the hybrid structure promotes the formation of a protective, char-like layer on the surface of the polymer during combustion. This char acts as a barrier, slowing down the diffusion of oxygen into the material and the escape of volatile degradation products [27].
- Free Radical Trapping: the hybrid structures act as free radical scavengers, which inhibit the chain-reaction degradation mechanism, leading to improved thermal oxidative stability [21,22,28,29,30,31,32].

Typical kinetic improvements in CNT/GN-filled epoxy systems include higher onset and maximum degradation temperatures, increased activation energy (as determined by the Ozawa–Flynn–Wall, Kissinger–Akahira–Sunose, and Friedman methods [33,34,35,36]), and a reduced oxidative mass-loss rate due to restricted oxygen diffusion. These enhancements reflect not only thermal effects but also underlying structural and electrical changes within the nanocomposite.

Electrical behavior in CNT/GN-reinforced polymers is governed by the formation of percolated networks. The same interconnected network that enables electron transport also influences thermal degradation. A continuous CNT/GN pathway improves heat dissipation, delays oxidation, and promotes a more uniform temperature distribution, thereby reducing localized polymer breakdown. Higher electrical conductivity generally indicates better dispersion, a lower percolation threshold, and more efficient CNT–graphene synergy. Consequently, improved conductivity correlates with higher activation energy and slower oxidative degradation. Conversely, low conductivity often signals filler agglomeration or weak interfacial bonding, which create morphological defects that act as oxidation initiation sites and reduce thermal stability.

Morphology links the electrical and thermal responses. The CNT/GN hybrid structure promotes network formation, with CNTs acting as one-dimensional bridges between graphene sheets to enhance connectivity. Dispersion quality is critical: well-dispersed fillers simultaneously improve thermal resistance and electrical conductivity. Strong polymer–nanocarbon interfacial interactions further restrict chain mobility and slow oxidation. In this work, TUNA imaging confirms a homogeneous distribution and continuous conductive domains, consistent with the observed kinetic improvements. In contrast, poor morphology—characterized by agglomerates, voids, or disconnected domains—facilitates oxygen diffusion, disrupts electrical pathways, and concentrates thermal stress. Thus, morphology governs the effectiveness of the carbon network, while the electrical and thermal properties reflect its quality.

When morphology, electrical behavior, and thermal degradation kinetics are considered together, the system can be described through a triangular structure–property–performance relationship. In this framework, the morphology of the CNT/GN hybrid network (dispersion, alignment, interfacial bonding) represents a structural feature that can influence the continuity and density of conductive pathways within the epoxy matrix. The resulting electrical properties (percolation threshold, conductivity, network density) therefore serve as indicators of the degree of network development rather than as mechanistic intermediates. The observed thermal degradation behavior (activation energy, onset temperature, oxidation rate) may be associated with these electrical characteristics, since more continuous hybrid networks are typically consistent with improved heat dissipation and enhanced barrier effects. Accordingly, a well-organized CNT/GN hybrid network is compatible with higher conductivity and, in turn, with thermal degradation profiles indicative of slower oxidative processes. These relationships should be interpreted as correlations within a conceptual framework rather than as strict cause-and-effect sequences.

## 2. Materials and Methods

The conductive nanofillers used in this study are multi-walled carbon nanotubes (CNTs) and graphene nanosheets (GNs). Catalytic carbon vapor deposition (CCVD) was employed by Nanocyl S.A (3100 Grade—Sambreville, Belgium) for the preparation of CNTs. Once CNTs exit the reactor, they were purified to reach a purity of over 95% by mass of carbon. The outer diameter and the length of the CNTs range from 10 to 30 and from 100 to 1000 nm, respectively. The Brunauer–Emmett–Teller method has allowed us to determine the specific surface area of CNTs, whose value is about 250–300 m^2^/g.

GNs (Asbury graphite grade 3759, Asbury Carbons, Asbury, NJ, USA) were synthesized via an intercalation/exfoliation process, whose precursor was natural graphite with 500 µm average diameter [37]. Highly irregularly shaped GN nanoparticles were found, containing graphitic moieties with a number of layers ranging from 5 to 29. Further details are available in a previous paper [38].

The epoxy matrix consists of the following materials: a mixture of tetraglycidyl methylene dianiline (TGMDA) and 1,4-butane-diol diglycidyl ether (BDE) in an 80:20 weight ratio. A curing agent, 4,4′-diaminodiphenyl sulfone (DDS), was incorporated at a stoichiometric level of 55 parts by weight for every hundred parts of the resin mix of TGMDA and BDE. These chemical substances were provided by Merck Life Science S.r.l. (Milan, Italy). Hybrid epoxy samples were formulated by combining the two nanofillers, CNTs and GNs, at two distinct weight percentages (0.1 and 0.5%) with epoxy precursors (TGMDA + BDE). It is important to mention that the chosen amounts (0.1 and 0.5%) represent the weight percentages below and above the Electrical Percolation Threshold (EPT) for the two binary polymer composites based only on CNTs or GNs nanofillers [38], respectively. Filler concentrations were explored in a range immediately below and above the electrical percolation threshold (EPT), since it is in this region that the formation of the conductive network is most sensitive to the morphology and combination of fillers. Below the EPT, the differences between hybrid and single-filler systems highlight the efficiency with which CNTs and graphene cooperate in reducing percolation. Above the EPT, the network is already formed, and a further increase in filler content quickly leads to a saturation regime, where the differences between hybrid and single-filler systems become insignificant. For this reason, concentrations too far from the EPT are not useful for evaluating the synergistic effect.

The so-obtained hybrid material samples were denoted as HYB_X%_Y:Z, where X% is the weight percentage of the total amount of both the fillers (0.1 or 0.5%, respectively), while Y and Z are the different weight ratios, CNTs:GNs, considered in this study: 5:1, 1:1 and 1:5, respectively, for a total of six hybrid nanocomposites. Table 1 summarizes all the correlations between the sample labels and the composition.

More specifically, the hybrid nanocomposites were prepared by incorporating and evenly distributing both the 1D and 2D nanofillers in the appropriate mix ratios within the epoxy mixture (TGMDA + BDE) at a temperature of 90 °C for 20 min, utilizing a Hielscher model UP200S (200 W, 24 kHz) ultrasound device from Hielscher Ultrasonics GmbH in Teltow, Germany. Following this, the temperature was increased to 120 °C, at which point the DDS was introduced into the blend and stirred magnetically until the crosslinking agent was fully dissolved. Next, the resulting nanofilled liquid formulations underwent a degassing process at approximately 100 °C under vacuum for about an hour to eliminate any bubbles caused by trapped air within the mixture. Finally, all the hybrid epoxy samples were cured in appropriate molds in an oven for one hour at 125 °C and then for three hours at 200 °C.

Several experimental techniques were used to characterize the hybrid nanomaterials, including thermogravimetry (TG), differential thermal analysis (DTA), and simultaneously (TG/DTA) operating. Thermal analyses (TG, DTA, TG/DTA) were performed according to ASTM E1131, ASTM E2550, and ISO 11358, which specify mass-based requirements (5–15 mg) rather than fixed specimen dimensions. More precisely, sample masses were prepared by cutting each of them into small pieces in order to have initially around 15 mg, precisely weighed.

Morphological and electrical characterizations of the formulated hybrid nanocomposites were carried out using the TUNA technique (BrukerNanoScopeV multimodeAFM, Digital Instruments, Santa Barbara, CA, USA), whose experimental setup is shown in the Supplementary Materials section (Figure S1). Nanoscale electrical characterization by TUNA follows instrument-specific AFM protocols, as no ASTM/ISO standard currently exists; samples were prepared as flat polished pieces (≈5–10 mm) suitable for AFM mounting.

The TG/DTA experiments were carried out with a Setaram SETSYS 1200 instrument (Mougins, France) under air flow at 50 mL/min at four different heating rates (2, 4, 7 and 10 K/min) to perform kinetic computations. Identical blank experiments with empty pans under identical operative conditions were recorded to adjust both the baselines of TG and DTA curves.

For kinetic computations, the TG data regarding the temperature ranges where the investigated processes occurred were processed using a common Microsoft Excel spreadsheet tool.

The data regarding the topography and local nano electric current of the hybrid nanocomposites were acquired using TUNA, which operates in contact mode, employing platinum-coated probes with nominal spring constants of 35 N m^−1^ and a conductive tip of 20 nm.

The TUNA module gauges ultra-low currents (<1 pA) from 80 fA to 120 pA flowing through the conductive tip to the samples being examined that are maintained at a constant DC bias. In this study, we applied a DC sample bias ranging from 1 V to 2 V.

A linear current amplifier within a range of 60–120 fA measures the resulting current flowing through the samples. In this manner, the sample’s topography and current are assessed simultaneously, allowing for a direct correlation between the sample’s position and its electrical characteristics.

It is important to mention that particularly sensitive current measurements are possible because the noise level of the TUNA module is typically around 50 fA.

The most detailed current mapping of the nanocomposites was achieved using the present sensitivity of the TUNA module, which configures the gain based on the TUNA sensor’s output voltage set to 1 pA/V, equating to a gain of 10^12^, a scan rate of 0.500 Hz s^−1^, and with the number of pixels in X and Y (samples/line) set to 512.

The areas identified in the examined samples are indicative of the full set of the hybrid nanocomposites, as a cantilever with a sharp tip scanned multiple regions on the sample surface to achieve electric measurements at the nanoscale level with guaranteed repeatability and reproducibility. Each TUNA image presented in the manuscript was taken after confirming that the electrical response was consistent across at least five different scanned locations.

It is important to mention that, in general, it is insufficient for the tip to merely touch a conductive material; effective electrical connections to the ground achieved with silver paste are crucial for current flow.

Therefore, a current signal is generated only if the tip during the sample contact forms part of a closed electrical circuit. In this study, the characterization at nanoscale level was performed without grounding the samples.

The TUNA findings indicate that, although the examined samples are ungrounded, it is feasible to identify electric current measurements that undeniably confirm the inherent electrical conductivity of the developed nanocomposites.

The TUNA images were examined utilizing the Bruker software Nanoscope Analysis 1.80 (Build R1.126200). To emphasize the morphological characteristics of the samples, their dispersion within the polymer matrix, and their interaction with the epoxy domains, the nanocomposites were subjected to an etching process before TUNA examination. The etching solution was created by mixing 1.0 g of potassium permanganate in a solution of 95 mL sulfuric acid (95–97%) and 48 mL of orthophosphoric acid (85%). The filled resins were submerged in the new etching solution at room temperature and stirred for 36 h. The following washes were carried out with a cold blend of two volumes of concentrated sulfuric acid and seven volumes of water. Subsequently, the samples were rinsed once more with 30% aqueous hydrogen peroxide to eliminate any manganese dioxide. The samples were ultimately rinsed with distilled water and stored in a vacuum for 5 days before undergoing morphological analysis.

To ensure reproducibility, all TG measurements were performed five times on independently prepared specimens, and the reported values represent the average of these replicates. TUNA analysis was conducted on five distinct regions per sample, and the measured current values were consistent across regions, confirming the spatial reproducibility of the conductive network. The TUNA maps shown correspond to the representative average behavior.

All samples were prepared using the same CNT/GN filler type and an identical dispersion protocol, which is known to produce stable and repeatable microstructures when processing conditions are unchanged. The resulting datasets exhibited low variability, confirming the robustness of the preparation method.

## 3. Theoretical Background of Kinetic Analysis

Experimental thermal analysis (TG or DTA) raw data collected at several heating rates in the appropriate temperature ranges where the oxidative thermal degradation takes place can be processed according to a well-known reliable model-free kinetic procedure aiming at determining kinetic parameters without a priori assumption on the reaction mechanism. The investigated process is often complex in nature and sometimes it occurs in some partially overlapping steps, causing a not negligible dependence of these parameters from the degree of conversion *α* = (*m*_i_ − *m*_T_)/(*m*_f_ − *m*_i_), where *m*_i_, *m*_f_, and *m*_T_ are the masses at the initial, final and temperature *T*, respectively, as determined from thermal analysis data.

Any kinetic computation carried out by processing thermal analysis data is based on the following general rate equation [39]:

d*α*/d*t* = *k*(*T*) *f*(*α*), (1)

where *k*(*T*) and *f*(α) are mutually independent functions of temperature and conversion, respectively, which may not have a real physical meaning.

Strictly speaking, *k*(*T*) is often written in the form of the Arrhenius equation, *k*(*T*) = *A*_k_ exp(−*E*/RT), being *A*_k_ and *E* the pre-exponential factor and activation energy, respectively. These Arrhenius parameters should depend only on temperature, but usually a small variation with conversion may be considered acceptable, and one of the most commonly used integral isoconversional methods (namely, the Kissinger–Akahira–Sunose (KAS)) [35]) may be adopted. If this condition (small variation in *E* values) is fulfilled, the activation energy *E* can be determined according to the KAS method at each constant degree of conversion from the slope of each regression line by plotting ln[*β*/(*T*α)^2^ against the reciprocal temperature, where *β* = d*T*/d*t*.

On the other hand, sometimes *k*(*T*) and *f*(α) have a physical meaning and for lifetime prediction purposes the temperature function can be effectively be expressed by a non-Arrhenian equation [40], since Arrhenius temperature dependence often lead to unrealistic overestimates of reaction times, especially if the extrapolated temperatures is largely outside the experimental temperature range explored [40,41]. More precisely, a non-Arrhenius *k*(*T*) temperature function was successfully adopted (in comparison with Arrhenius one) to accelerated thermooxidative tests of different polyolefines and other polymers and reaction times extrapolated to low service temperatures was realistic from the former and unrealistically high for the latter [41]. Arrhenian behavior is particularly appropriate for describing chloroprene rubber cable jacketing materials [42] as well as for the oxidative thermal degradation of epoxy/CNT–graphene hybrid composites here investigated, due to the intrinsically multi-step and conversion-dependent nature of their processes. The overlapping contributions of matrix scission, char formation, and oxidation of carbonaceous residues violate the fundamental Arrhenius assumption of a single mechanism with constant activation energy. As widely reported for filled thermosets and char-forming polymer systems, enforcing an Arrhenius temperature dependence often leads to non-physical activation energies and, most critically, to unrealistic reaction-time predictions, which represent the key kinetic parameter for assessing the thermal endurance of these materials.

In contrast, the incremental method based on a non-Arrhenian *k*(*T*) temperature function does not impose a predefined reaction model and, at the same time, does not assume a physically meaningful description of the mechanistic transitions induced by CNT/GN hybrid networks. This results in more reliable and experimentally consistent lifetime estimates, in agreement with previous studies demonstrating that non-Arrhenius treatments provide significantly more realistic reaction times for heterogeneous nanocomposite systems. The improved coherence between kinetic parameters and the experimental TGA/DTG profiles confirms the suitability and advantage of the non-Arrhenius approach for the present hybrid composites.

A more realistic prediction can be obtained using other *k*(*T*) functions, like the Berthelot–Hood equation [43]:

*k*(*T*) = *A*′_k_ exp(*DT*), (2)

where *A*′_k_ and *D* are adjustable parameters. From thermal analysis experiments under constant *β* values, by combining Equations (1) and (2) and by integrating within a small conversion increment α_i+1_ − α_i_, it yields:

1 = [exp(*DT*_i_) − exp(*DT*_i−1_)]/*A*′*D β* (3)

where *T*_i−1_ and *T*_i_ are the temperatures corresponding to the degree of conversion values α_i+1_ and α_i_, respectively, and *A*′ = [g(α_i_) − g(α_i–1_)]/*A*′_k_, being g(α) the integral conversion function, equal to 1/*f*(α). Equation (3) provides a regression model that fits experimental raw data according to a non-linear square method. From each α(*T*) vs. *T* plot recorded at each given heating rate, the so-called isoconversional temperature is evaluated at predefined conversion levels (every 0.05 segment in this study), while the conversion function *f*(α) is obviously constant.

Rewriting the left hand-side of Equation (1) in the form dα/d*t* = (dα/d*T*) · *β*, after separation of the two variables and integration, with *k*(*T*) according to Equation (2), at each constant degree of conversion two distinct values of *A*′ and *D* can be determined from the intercept and the slope of each *i* regression line:

ln[*β*/(*T*_i_ − *T*_i−1_)] = −ln *A*′_i_ + *D*_i_⋯(*T*_i−1_ − *T*_i_)/2 (4)

obtained by plotting the left-hand side of Equation (4) against (*T*_i−1_ − *T*_i_)/2.

Finally, the set of *A*′_i_ and *D*_i_ pairs were used to determine the reaction time interval ∆*t*_i_ to achieve the variation in the degree of conversion from α_i–1_ to α_i_ at a given temperature *T* via the following Equation (5):

∆*t*_i_ = (*t*_i_ − *t*_i−1_) = *A*′_i_⋯exp(−*D*_i_*T*)/2 (5)

The goodness of this prediction through this kinetic model is made by comparing the predicted α(*t*) curves at given reasonably low constant temperatures with the reconstructed ones by summing all time increments calculated via Equation (5).

## 4. Results and Discussion

### 4.1. Thermal Behavior Study

**Figure 1:**
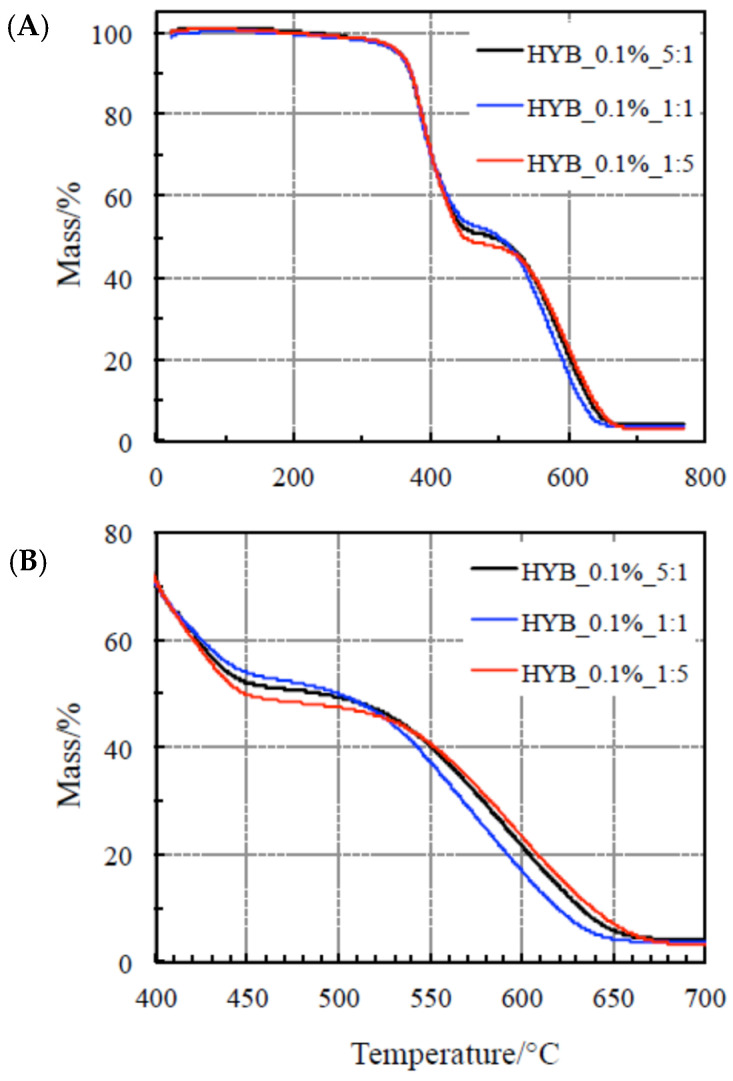
(**A**) TG of the nanocomposites with 0.1% hybrid nanocharge (HYB_0.1%_CNTs:GNs) and (**B**) details of the TG of the same samples for the second step only.

**Figure 2:**
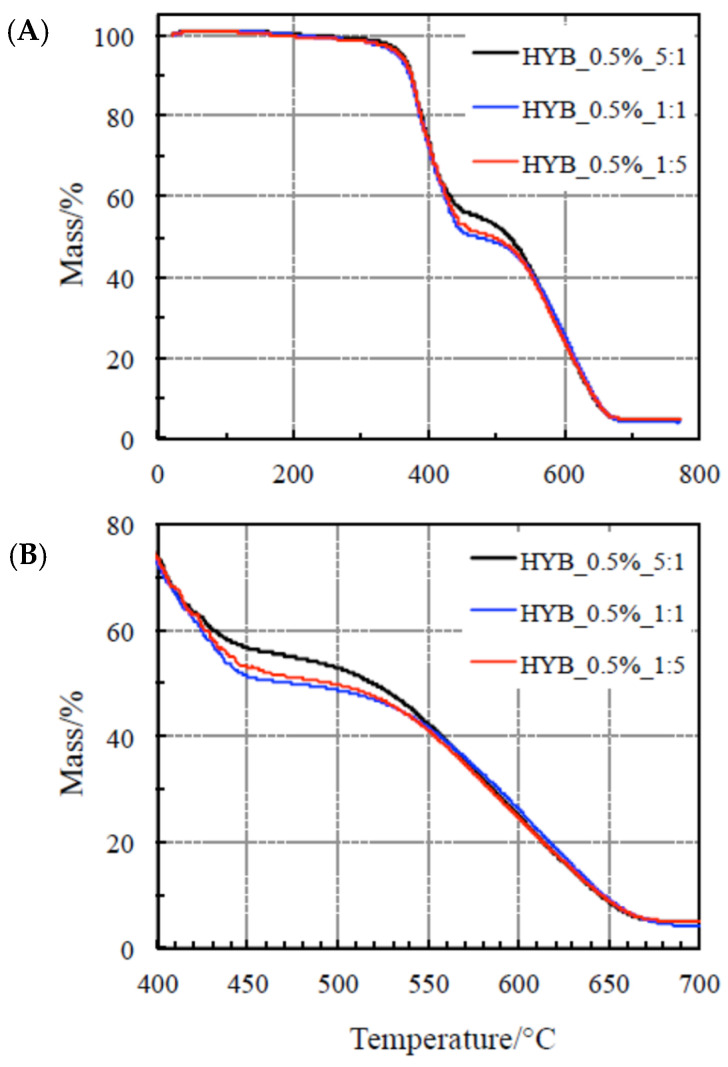
(**A**) TG of the nanocomposites with 0.5% hybrid nanocharge (HYB_0.5%_CNTs:GNs) and (**B**) details of the TG of the same samples for the second step only.

The thermal behavior of the two polymer composite series with the two total amounts of mixed fillers CNTs and GNs (0.1 and 0.5% by weight) is studied by examining the corresponding TG curves carried out under a flowing air atmosphere up to about 800 °C. The plots are reported in Figure 1 and Figure 2, respectively. All these polymer composites exhibit a remarkable stability with no mass loss up to 200 °C.

**Figure 3:**
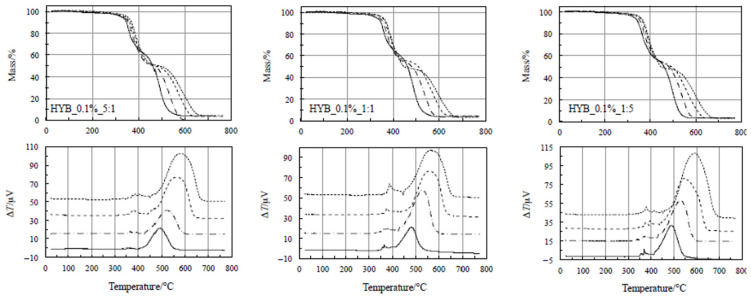
TG/DTA at various heating rates for the nanocomposites with 0.1% hybrid nanocharge (HYB_0.1%_CNTs:GNs).

**Figure 4:**
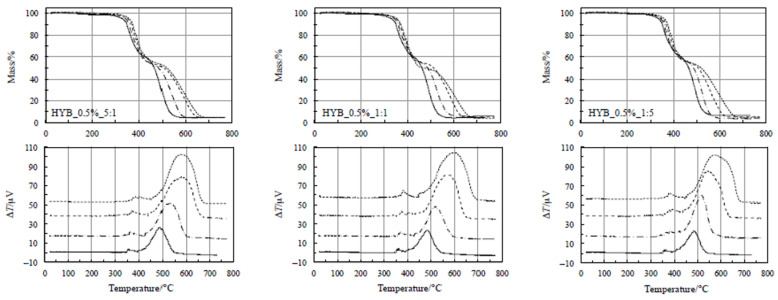
TG/DTA at various heating rates for the nanocomposites with 0.5% hybrid nanocharge (HYB_0.5%_CNTs:GNs).

At higher temperatures, two evident steps occurred, exothermic in nature. The former takes place between 200 and 470 °C and is accompanied by a small heat release (as clearly visible in Figure 3 and Figure 4). According to a previous study reported in the literature [37,38,44,45,46,47], this step is ascribed to a crosslinking process, as confirmed by Dynamic Mechanical Analysis (DMA) [38]. Negligible differences are clearly visible among the TG curves for this process, so that this change in the mechanical properties seems not to be affected by the total amount of fillers, nor by the relative amount of CNTs and GNs in the samples. In other words, no reasonable conclusions can be drawn on the thermal stabilities of the polymer composites on the basis of this process. Figure 3 and Figure 4 show that the shift toward higher temperatures by increasing the heating rate for all the samples is also negligible, thus suggesting that this process could be quite fast (with low activation energy values).

A very intense exothermic DTA peak is associated with the second step, between 470 and 700 °C, with a corresponding mass loss of about 50 mass%. A final residue of about 4% is detected at the end of each TG experiment. On the basis of a previous study [38], because of the presence of an air environment, this process is attributed to the oxidative decomposition. As far as the second step is concerned, Figure 3 and Figure 4 show that the shift toward higher temperatures with increasing the heating rate for all the samples is more evident, thus concluding that the reaction rate is lower. As a consequence, this process is more suitable (than the first one) to be considered for assessing the nanocomposites’ thermal stability through the kinetic computations, although this step occurs at higher temperatures, since this is the rate-determining step.

### 4.2. Kinetic Analysis of Thermal Decomposition and Assessment of Thermal Stability

Once the thermal behavior of the hybrid composites has been examined, we mainly attempted to assess their thermal stability by analyzing the kinetics of the second step of mass loss in the TG curves, namely the oxidative decomposition. Kinetic analysis was restricted to the second degradation stage, as this step corresponds to the well-defined oxidative decomposition of the char, whereas the first stage involves overlapping pyrolytic events that do not allow reliable isoconversional evaluation. More precisely, the portions of the TG curves at different heating rates related to the first step (upper plots of Figure 4) are almost superimposable and the isoconversional temperatures (*T* at the same degree of conversion for different heating rates) do not always increase with increasing the heating rate, as expected, leading to a less reliable kinetic model, especially if data is extrapolated to low temperatures below the experimental ones. Furthermore, a quantitative evaluation of the DTG curves indicates that the first degradation stage contributes only ~20–30% of the total mass loss, whereas the second stage accounts for ~70–80% across all formulations. These aspects confirm the dominant role of the second stage in the overall decomposition process and justifies focusing the kinetic analysis on this region, where the oxidative degradation of the carbonaceous char occurs and the activation energy values are physically meaningful. A first preliminary approach was carried out using the KAS method, according to what we did in some previous studies [48,49].

**Figure 5:**
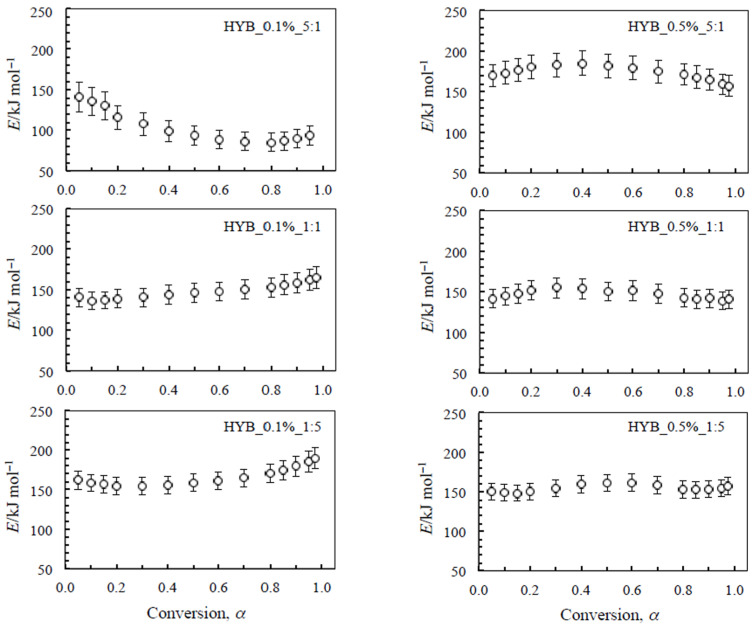
Isoconversional (*E* vs. α) plot of the second step (i.e., the oxidative decomposition) for the nanocomposites with 0.1% and 0.5% hybrid nanocharge (HYB_0.1%_CNTs:GNs and HYB_0.5%_CNTs:GNs, respectively).

The isoconversional plot of this process for all nanocomposites tested is shown in Figure 5. Taking into account also the associated uncertainties (reported as errors bars that do not exceed 13% of the *E* mean value for each plot), a negligible variation in activation energy is observed with increasing the degree of conversion, except for the process related to HYB_0.1%_5:1, for which a detectable decrease in *E* values (from 142 to 96 kJ mol^−1^ up to α = 0.7) is calculated.

As a preliminary evaluation of lifetime prediction, for the remaining five samples, practically constant *E* values were found with the increase in the degree of conversion. Therefore, if we attribute to this parameter the common physical meaning of an energy barrier to be overcome to obtain the products, some considerations can be deduced.

First, for the two composites with lower filler mix content (0.1%), a slightly higher stability (due to a higher energy barrier) seems to be ascribable to the sample with the higher content of GNs (HYB_0.1%_1:5). In this regard, it is worth noting that at 0.1% the conductivity maximum (1:1) and the thermal stability maximum (1:5) do not coincide because electrical transport near percolation is governed by network connectivity, whereas oxidative stability depends on the graphene-rich char structure and its barrier efficiency. Thus, the two properties probe different mechanisms at sub-percolative loading.

Second, the highest energy barrier and consequently the highest stability seem to be attributable to the sample with the richest total amount of filler mix (0.5%) and the lowest amount of GNs (HYB_0.5%_5:1).

The differences are of the same magnitude order as the associated uncertainties, so that in this case, this approach based on the evaluation of activation energy cannot be considered appropriate to assess a final thermal stability scale. A better stability parameter is the reaction time to reach a given conversion degree at a defined temperature, usually extrapolated as lower temperature than the experimental ones (Equation (5)). To achieve this goal and to obtain a more reliable and realistic lifetime prediction, several authors [41,42] suggested using the incremental method, based on the previously mentioned Equations (2)–(5).

**Figure 6:**
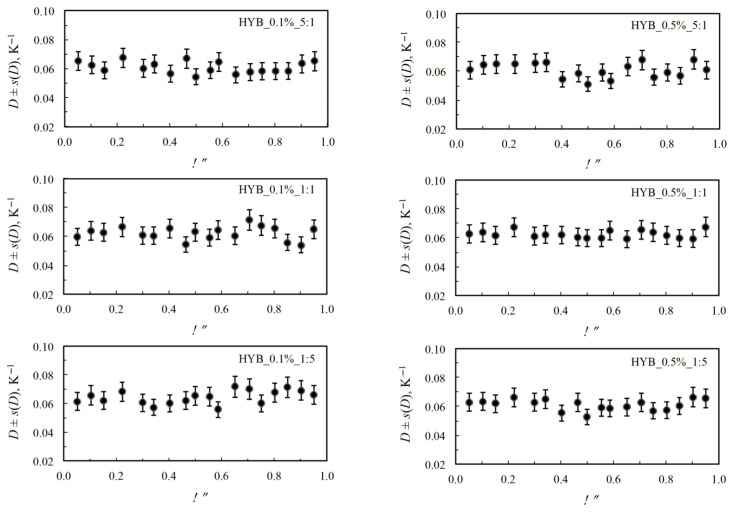
Non-Arrhenian *D* values as a function of the degree of conversion for the second stage of the oxidative decomposition for nanocomposites with 0.1% and 0.5% hybrid nanocharge (HYB_0.1%_CNTs:GNs and HYB_0.5%_CNTs:GNs, respectively).

For the second step of mass loss of all the composite samples, the *A*′ and *D* parameters, at each fixed degree of conversion, are respectively calculated from the intercept and the slope of the regression line obtained by plotting the left-hand side of Equation (4) versus (*T*_i−1_ − *T*_i_)/2. The isoconversional *D* values, corresponding to each conversion range Δα = α_i−1_ − α_i_, and the associated uncertainties (as error bars) are reported in Figure 6.

The values of *D* seem to be practically constant, and only negligible random variation are observed (within the estimated uncertainties) as a function of the degree of conversion.

Lifetime can be predicted by calculating the reaction time interval ∆*t*_i_ (expressed in years) to achieve a degree of conversion up to 0.20 at a given reasonably low temperature *T* (310.15, 323.15 and 353.15 K), according to Equation (5).

The reaction time intervals are reported in Table 2 with estimated errors that do not exceed 7% at the lowest extrapolated temperature. At each constant temperature, for a given degree of conversion, it can be observed that the longer the reaction time interval, the greater the thermal stability. So, looking at the extrapolated tα values in Table 2, it seems evident that the thermal stability of the poor-filler nanocomposites (having 0.1% of the total amount of mixed fillers CNTs and GNs) increases with the increase in the amount of GNs (HYB_0.1%_1:5), while for rich-filler nanocomposites (having 0.5% of the total amount of mixed fillers CNTs and GNs), the most stable material is that with the lower content of GNs (HYB_0.5%_5:1). In this regard, at 0.5% mixed filler total loading, the superior thermal stability of CNT-rich formulations can be rationalized by considering the different microstructural architectures formed by CNTs and GNs. CNTs generate more continuous and interconnected pathways, enabling efficient phonon transport and reducing the likelihood of localized thermal accumulation. In contrast, the excessive stacking tendency of GNs at low loadings promotes the formation of compact agglomerates that behave as micro-defect sites and interrupt heat-dissipation routes. Although quantitative metrics such as porosity or interfacial-bonding strength were not directly measured, the combined evidence from electrical conductivity, percolation behavior, and morphological analyses supports this mechanistic interpretation. The absence of porosity-related features in the micrographs further indicates that the observed differences in thermal stability arise from network topology rather than from void formation or interfacial degradation.

### 4.3. Morphological and Electrical Analyses by TUNA

In this study, the analysis is focused on the hybrid CNT/GN epoxy composites, as the behavior of the individual filler systems (CNT-only and GN-only) has already been extensively characterized in our previous paper [50]. In that study, the morphological, electrical, and computational responses of the single-filler composites were thoroughly investigated, demonstrating the synergistic effect of CNTs and GNs on the electrical properties through the correlation between molecular-scale simulations and DC conductivity measurements. These results provide a validated baseline for the individual fillers, which is used here as the reference framework to isolate the additional effects arising uniquely from the CNT/GN hybrid network. For this reason, the preparation of new control samples containing only CNTs or only GNs was not repeated, allowing the present work to focus specifically on the hybrid formulations and their enhanced functional response. The correlation between electrical properties and thermal stability in CNT/GN-filled polymer composites shows that a well-connected CNT/GN network creates a continuous conductive path. This enhances heat dissipation and slows oxidation. Higher electrical conductivity leads to better dispersion and a lower electrical percolation threshold (EPT), boosting thermal stability and reducing oxidative degradation. Tunneling Atomic Force Microscopy (TUNA) technique is effective for studying the nanoscale structure and charge-transport pathways in these hybrid nanocomposites, allowing simultaneous mapping of morphology and ultra-low electrical currents in epoxy nanocomposites reinforced with carbon nanofillers [37,38,47,50,51,52].

**Figure 7:**
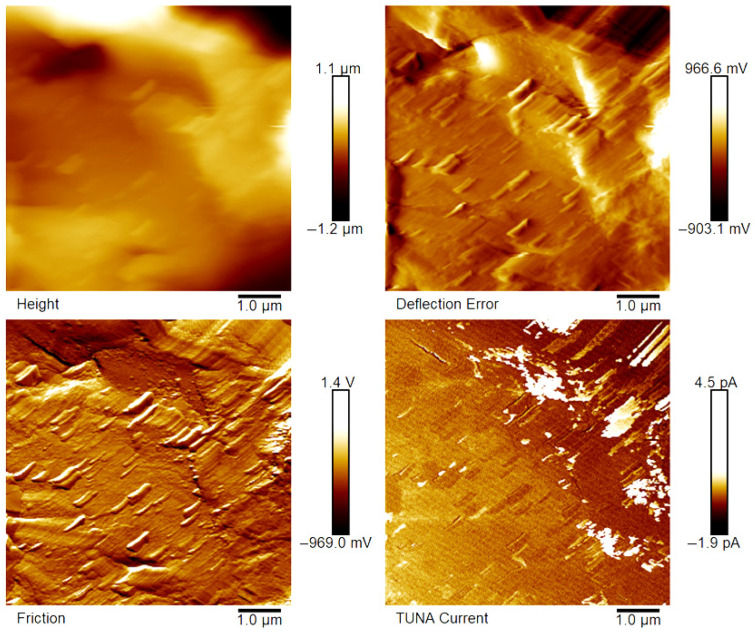
TUNA images of epoxy hybrid HYB_0.1%_5:1.

**Figure 8:**
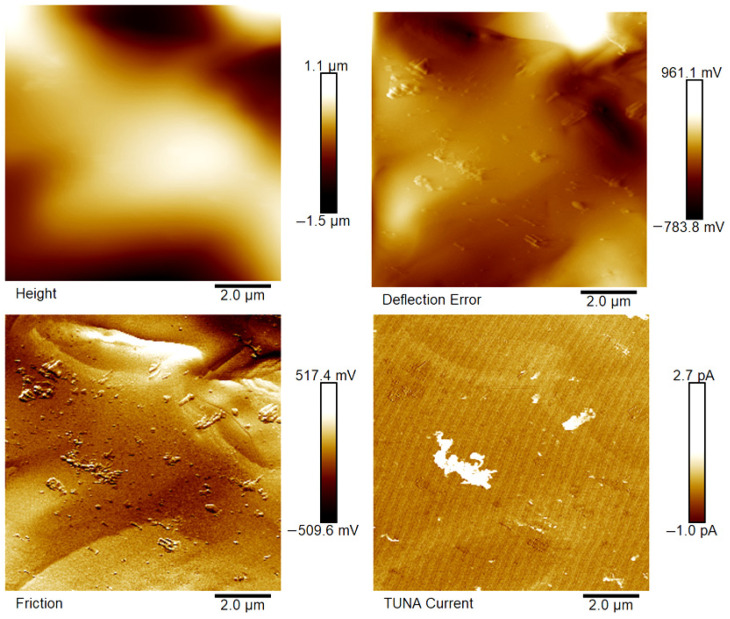
TUNA images of epoxy hybrid HYB_0.1%_1:1.

**Figure 9:**
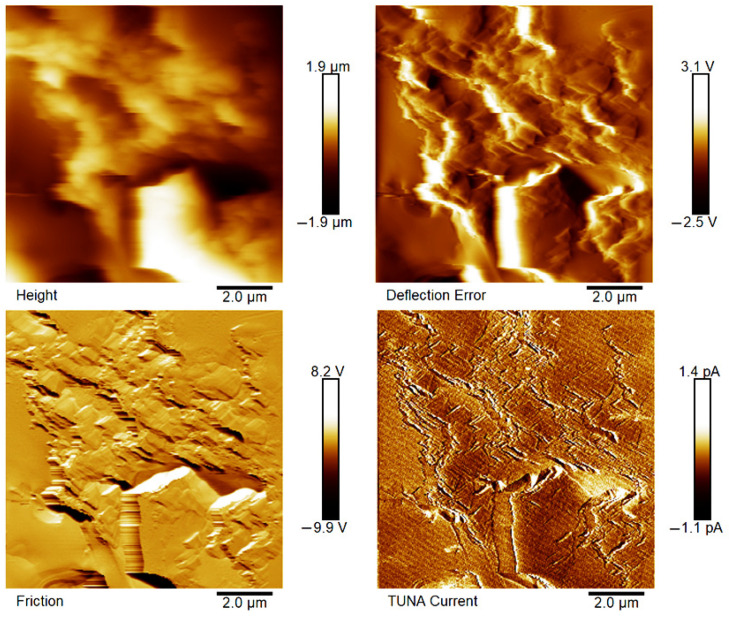
TUNA images of epoxy hybrid HYB_0.1%_1:5.

**Figure 10:**
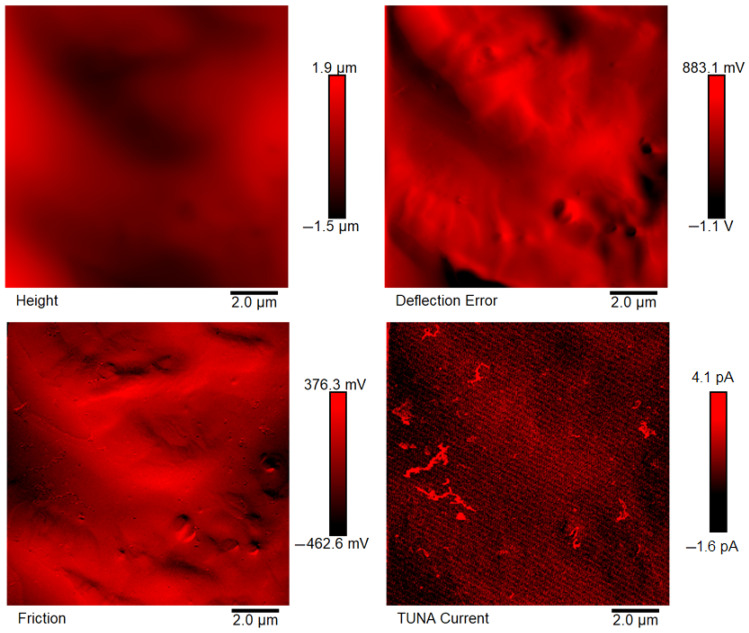
TUNA images of epoxy hybrid HYB_0.5%_5:1.

**Figure 11:**
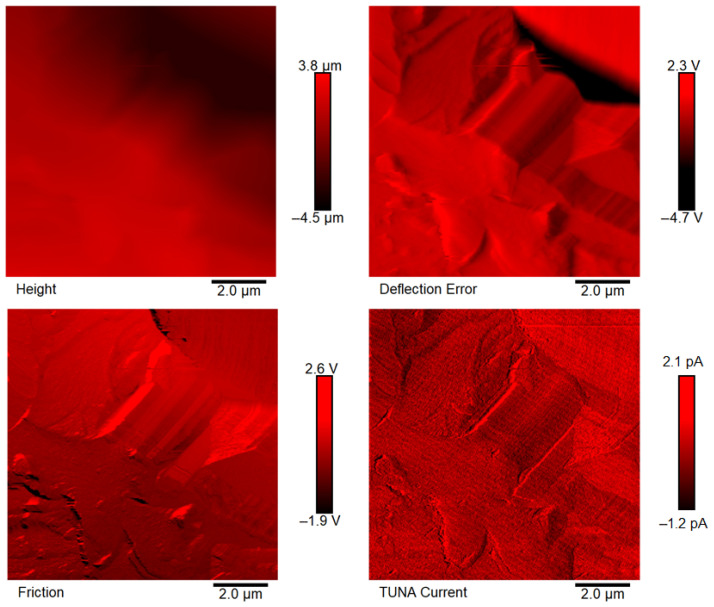
TUNA images of epoxy hybrid HYB_0.5%_1:1.

**Figure 12:**
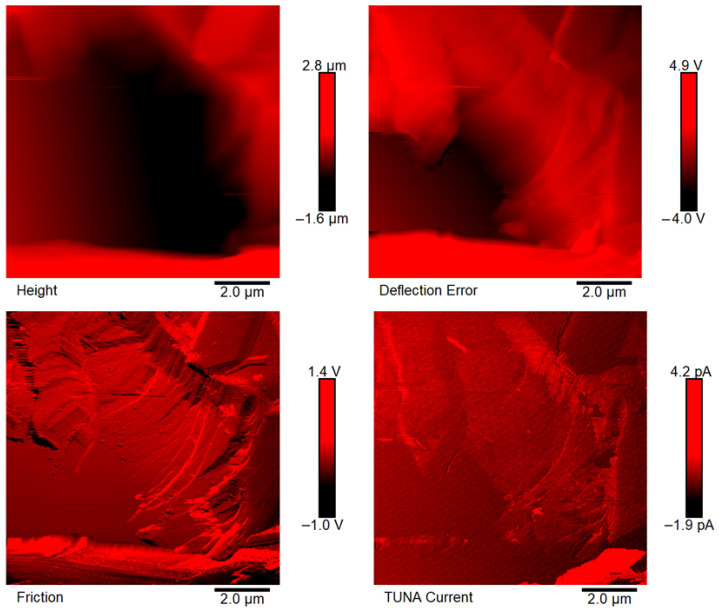
TUNA images of epoxy hybrid HYB_0.5%_1:5.

Figure 7, Figure 8, Figure 9, Figure 10, Figure 11 and Figure 12 display four types of TUNA images for six hybrid samples: HYB_0.1%_5:1, HYB_0.1%_1:1, HYB_0.1%_1:5, HYB_0.5%_5:1, HYB_0.5%_1:1, and HYB_0.5%_1:5. For each sample, Height, Deflection Error, Friction, and TUNA Current images were collected at the same time, allowing for analysis of morphology, material differences, and electrical properties at the nanoscale. The height image (topography) measures the actual surface topography from the TUNA scanner’s movements. Each pixel indicates the height needed to keep the feedback set-point during scanning, revealing surface roughness, nanofiller distribution, matrix–filler interfaces, and features of the materials. In hybrid nanocomposites, the height image shows CNT bundles, graphene nanosheets, and surface texture changes due to π–π interactions in carbon nanostructures [50], serving as a reference for interpreting other signals (mechanical or electrical). Deflection Error image measures the difference between actual cantilever deflection and the feedback setpoint, providing a high-contrast map of surface features. It reveals details about sharp edges and small morphological features in hybrid nanocomposites, highlighting CNT edges, graphene wrinkles, and polymer matrix heterogeneities. Friction Image measures cantilever torsional twist, reflecting variations in surface chemistry and mechanical properties. It identifies differences in friction between the polymer matrix and CNTs or graphene, helping to distinguish material contrast and orientation of nanofillers affected by oxidation.

TUNA Current Image measures ultra-low electrical currents (pA–fA) between a conductive tip and a sample under bias, revealing the spatial distribution of electrically active regions such as conductive CNT networks, graphene platelets, junctions between fillers, and local breakdown of conductive pathways. It indicates charge-transport efficiency and network connectivity, aiding in understanding percolation thresholds and synergistic effects in CNTs and graphene. Morphological characterization with TUNA assesses conductive pathways and filler dispersion in CNTs/GNs hybrid nanocomposites.

Prior work [38,50] shows that carbon nanotubes (CNTs) and graphene nanosheets (GNs) synergistically enhance the mechanical, electrical, and thermal properties of hybrid materials. CNTs act as bridges between the GNs, increasing the contact area with the polymer matrix and reducing GN aggregation. Key π–π interactions and strong interfacial bonds lead to improved performance and conductivity compared to materials with single fillers, forming a cross-linked network that optimizes properties. Measurements of direct current (DC) conductivity on hybrid nanocomposites demonstrated significant increases in electrical conductivity with the inclusion of graphene nanosheets (GNs) at 0.1% of total mass amounts of the mixed fillers [50]. In fact, the mix ratios CNTs:GNs (5:1, 1:1, 1:5) yielded conductivities of 2.49 × 10^−4^ S/m, 3.73 × 10^−4^ S/m, and 3.81 × 10^−6^ S/m, respectively, which are significantly higher than the value of 1.06 × 10^−13^ S/m measured for the sample loaded with 0.1% of CNTs alone, which has an insulating behavior. An epoxy sample with only 0.1% GNs had a conductivity of 2.48 × 10^−5^ S/m, illustrating graphene’s efficacy in enhancing electrical properties. It is worth noting that 0.1%, is a quantity lower than the EPT observed with mono-loaded epoxy systems [50]. At 0.5%, hybrid nanocomposites showed conductivities on par with mono-component CNTs/system (1.55 × 10^−2^ S/m) and GNs/system (4.18 × 10^−2^ S/m). The highest values observed were 3.04 × 10^−2^ S/m for 1:5 and 2.94 × 10^−2^ S/m for 5:1, although a high value of 9.79 × 10^−3^ S/m was also recorded for the 1:1 mix ratio. These findings suggest a diminished synergistic effect of mixing at higher filler concentrations where an established conductive network exists [50]. The morphological features of the two nanofillers, CNTs and GNs, utilized to prepare the hybrid composites, their unique interactions, and their distribution in the polymer matrix were examined using TUNA (refer to Figure 7, Figure 8, Figure 9, Figure 10, Figure 11 and Figure 12). It is worth noting that a thorough and precise morphological analysis of the hybrid samples, aimed at reliable visualization of the conductive network formed by the carbon nanofillers, was enabled by a selective etching procedure, which facilitated the consumption of the resin and revealed the two nanofillers. This treatment, already consolidated in our published papers [37,38,50], removes the amorphous fraction of the resin that would otherwise mask the percolation pathways and reduce the sensitivity of the TUNA signal. The morphology of the nanofillers exposed after etching is consistent with what was observed on untreated surfaces using complementary techniques, indicating that the process does not alter the structure of the conductive network but allows for clearer identification. TUNA images of epoxy hybrid HYB_0.1%_5:1 (Figure 7) depict stacked graphene nanosheets connected to carbon nanotubes, which are firmly attached to the host matrix. Carbon nanotubes (CNTs) served as a link between the epoxy matrix and the graphene nanosheets (GNs) because they simultaneously linked both the matrix and GNs, enabling the composite to fill the void and transition to a conductor. Figure 7 shows that incorporating a minor amount of GNs (20%) enabled a synergistic effect in the HYB_0.1%_5:1 sample via the π–π bond interactions formed between the CNTs and the GNs, clearly detectable also in Figure S2 of the Supplementary Materials section which shows the Field Emission Scanning Electron Microscopy (FESEM) image of the epoxy hybrid HYB_0.1%_5:1. These connections influence the transmission of the inherent electrical conductivity of the two nanofillers to the matrix, thereby creating a highly cross-linked conductive network, also promoting the formation of conductive pathways that lead to an enhancement in the electrical performance of the resulting composite. The morphological characteristics of carbon-based nanofillers are clearly essential for producing electrically conductive channels within the developed hybrid composites. In Figure 7, the carbon nanotubes present at a high percentage (80%) are uniformly distributed over the entire analyzed surface. They almost appear to orient themselves at the interface with the graphene nanosheets, with which they establish effective interactions also present in Figure 8 and Figure 9 where the formation of a cross-linked conductive network explains the high electric current values at the nanoscale detected by TUNA Current images. The values of electric current at the nanoscale are depicted in the TUNA Current image along the side scale bar, with colors transitioning from the darkest to the lightest, which correlate to regions of the sample exhibiting lower or higher electrical conductivity. We can observe a trend in the electric current values at nanoscale (from −1.9 pA to 4.5 pA for HYB_0.1%_5:1, from −1.0 pA to 2.7 pA for HYB_0.1%_1:1, from −1.1 pA to 1.4 pA for HYB_0.1%_1:5) that is perfectly correlated with that detected on a macroscale [46], namely 2.49 × 10^−4^ S/m, 3.73 × 10^−4^ S/m, 3.81 × 10^−6^ S/m, respectively. The presence of effective conductive pathways, attributed to well-dispersed nanofillers and strong interfacial interactions between the nanofillers and the host matrix, enables the formation of a highly cross-linked conductive network that ensures efficient electric charge transfer throughout the surface of the samples; this is also clearly seen in all epoxy samples with 0.5% hybrid nanofillers at the three CNTs:GNs ratios of 5:1, 1:1, and 1:5 (see Figure 10, Figure 11 and Figure 12). Even for these last epoxy hybrids, a trend can be noted in the electric current values at nanoscale (ranging from −1.6 pA to 4.1 pA for HYB_0.5%_5:1, from −1.2 pA to 2.1 pA for HYB_0.5%_1:1, from −1.9 pA to 4.2 pA for HYB_0.5%_1:5) that is completely aligned with what has been detected on a macroscale [46], specifically 2.94 × 10^−2^ S/m, 9.79 × 10^−3^ S/m, 3.04 × 10^−2^ S/m, respectively.

The π–π bond interactions between the CNTs and the GNs are also evident in Figure S3 of the Supplementary Materials section which shows the Field Emission Scanning Electron Microscopy (FESEM) image of the epoxy hybrid HYB_0.5%_5:1.

It is well established that the interfacial compatibility between carbon nanotubes, graphene nanosheets and the epoxy matrix governs not only the onset of oxidative thermal degradation but also the efficiency of charge transport and the formation of conductive pathways. As highlighted in recent studies [53], stronger polymer–nanofiller interactions promote more homogeneous dispersion, suppress filler–filler aggregation and enhance the stability of the hybrid network. In our system, the observed trends in thermal and electrical behavior are therefore consistent with the different interfacial affinities of CNTs and GNs: CNT-rich formulations benefit from a more continuous and resilient percolative architecture, whereas excessive GN content tends to increase restacking and reduce the effective interfacial contact area. These considerations support the mechanistic interpretation provided and rationalize the superior performance of CNT-dominated hybrid composites at low filler loadings.

## 5. Conclusions

Two different series of nanocomposites were prepared with multi-walled carbon nanotubes (CNTs) and graphene nanosheets (GNs) mixed together with 0.1 and 0.5 mass% of the total amount. In each series, three different CNT:GN mixing mass ratios, 5:1, 1:1, and 1:5, were considered. The thermal behavior and the surface area were determined by TG and DTA as well as by the Brunauer–Emmett–Teller method, while the electrical characterization was carried out by TUNA, aiming at evaluating the correlation among morphology, thermal behavior and kinetics of oxidative decomposition with the electrical properties of the hybrid nanocomposites.

One key focus of this study is to link the results of the kinetic analysis of oxidative degradation with the amounts of CNT and GNP in the epoxy-based composites at two different mass percentages of mixed fillers (0.1 and 0.5%). The research examines the reaction time for the second step of oxidative decomposition, which is influenced by factors like activation energy, morphology, and filler content. Findings indicate that increasing filler content enhances stability, as shown by increased reaction times at given conversion levels, which suggests better resistance to degradation. At constant temperature, a longer reaction time interval corresponds to greater thermal stability for a specific conversion degree, indicating slower oxidation kinetics. Extrapolated tα values indicate that poor-filler nanocomposites (0.1% mixed fillers) exhibit increased thermal stability with more GNs (HYB_0.1%_1:5), while rich-filler nanocomposites (0.5% mixed fillers) show highest stability with lower GNs content (HYB_0.5%_5:1). Summing up, the extrapolated *tα* values reveal an inversion in the stabilizing role of GNs: at 0.1% total filler, GN-rich hybrids enhance thermal stability due to improved dispersion and barrier effects, whereas at 0.5% loading, excessive GNs promote aggregation and disrupt CNT percolation, making CNT-rich hybrids thermally more robust.

Longer reaction times correspond with better dispersion and conductivity, forming a protective carbon network that acts as a barrier to oxygen diffusion. Evaluating reaction times at varying filler loadings helps to understand the trends in electrical properties at the nanoscale. Reaction time in the oxidative degradation process signifies how quickly oxidative damage begins. Higher reaction times at lower conversion indicate slower progression of degradation and greater material protection. Effective dispersion of hybrid fillers increases reaction times compared to neat epoxy, showing that a well-connected network can slow the onset of oxidative degradation.

The electrical properties of CNTs and GNP-filled polymers are influenced by percolation networks, which also interact with thermal degradation. Higher conductivity detected for the analyzed hybrid samples indicates better dispersion due to the strong interfacial bonding and effective network synergy between the nanofillers, leading to improved thermal stability. We can conclude that the morphological properties investigated by TUNA play a significant role in bridging the effects of thermal kinetics and electrical behavior.

## Figures and Tables

**Table 1:** Composition of the hybrid nanocomposites loaded with both 0.1 and 0.5 wt% of mixed fillers.

Sample Label	CNTs + GNs wt%	CNTs/ wt%	GNs/ wt%
HYB_0.1%_5:1	0.1	80	20
HYB_0.1%_1:1	0.1	50	50
HYB_0.1%_1:5	0.1	20	80
HYB_0.5%_5:1	0.5	80	20
HYB_0.5%_1:1	0.5	50	50
HYB_0.5%_1:5	0.5	20	80

**Table 2:** Reaction time interval values *t*_α_ estimated for the second step of the oxidative decomposition process of hybrid nanocomposites for a low degree of conversion. Uncertainties associated with *t*_α_ values do not exceed 4% at 353.15 K and 7% at 310.15 K.

α	HYB_0.1%_5:1	HYB_0.1%_1:1	HYB_0.1%_1:5	HYB_0.5%_5:1	HYB_0.5%_1:1	HYB_0.5%_1:5
	Reaction Time, *t*_α_/Years					
	310.15 K					
0.05	15.2	16.4	17.7	18.0	14.9	16.1
0.10	15.4	16.5	17.8	18.1	15.0	16.2
0.15	15.5	16.6	17.9	18.3	15.1	16.3
0.20	15.7	16.7	18.0	18.4	15.2	16.4
	323.15 K					
0.05	7.5	8.6	9.7	11.5	7.2	8.4
0.10	7.6	8.7	10.0	11.6	7.2	8.5
0.15	7.7	8.8	10.1	11.8	7.4	8.5
0.20	7.8	8.9	10.2	11.9	7.4	8.6
	353.15 K					
0.05	2.9	3.8	5.2	5.7	2.8	3.9
0.10	3.1	3.9	5.4	5.8	2.8	4.0
0.15	3.2	4.0	5.5	5.9	2.9	4.1
0.20	3.3	4.2	5.6	6.0	3.0	4.1

## Data Availability

The original contributions presented in this study are included in the article/Supplementary Material. Further inquiries can be directed to the corresponding authors.

## Supplementary Materials

The following supporting information can be downloaded at: https://www.mdpi.com/article/10.3390/polym18101150/s1, Figure S1: Experimental setup of TUNA instrument for acquiring topography and conductivity images simultaneously.; Figure S2: Field Emission Scanning Electron Microscopy (FESEM) image of the epoxy hybrid HYB_0.1%_5:1. (FESEM, mod. LEO 1525, Carl Zeiss SMT AG, Oberkochen, Germany).; Figure S3: Field Emission Scanning Electron Microscopy (FESEM) image of the epoxy hybrid HYB_0.5%_5:1. (FESEM, mod. LEO 1525, Carl Zeiss SMT AG, Oberkochen, Germany).

## References

[1] Cheng Y., Wang J., Shao M., Liang Y., Li H. (2025). Hybrid combinations of graphene nanoplatelet, carbon nanotube, and carbon black for tailored mechanical and triboelectric properties in polypropylene fibers. Adv. Compos. Hybrid Mater..

[2] Tang Z.-H., Wang D.-Y., Li Y.-Q., Fu S.-Y. (2026). Numerical investigation of synergistic enhancement of carbon nanotubes and graphene nanoplatelets on electrical properties of hybrid composites. Compos. Sci. Technol..

[3] Maxian O., Pedrazzoli D., Manas-Zloczower I. (2015). Modeling the electrical percolation behavior of hybrid nanocomposites based on carbon nanotubes and graphene nanoplatelets. Mater. Res. Express.

[4] Sagalianov I., Vovchenko L., Matzui L., Lazarenko O. (2017). Synergistic Enhancement of the Percolation Threshold in Hybrid Polymeric Nanocomposites Based on Carbon Nanotubes and Graphite Nanoplatelets. Nanoscale Res. Lett..

[5] Li H., Wang N., Han X., Yuan H., Xie J. (2021). Mechanism Identification and Kinetics Analysis of Thermal Degradation for Carbon Fiber/Epoxy Resin. Polymers.

[6] Bifulco A., Varganici C.D., Rosu L., Mustata F., Rosu D., Gaan S. (2022). Recent advances in flame retardant epoxy systems containing non-reactive DOPO based phosphorus additives. Polym. Degrad. Stab..

[7] Bao X., Wu F., Wang J. (2021). Thermal Degradation Behavior of Epoxy Resin Containing Modified Carbon Nanotubes. Polymers.

[8] Srivastava J., Gaur A. (2021). A tight-binding study of the electron transport through single-walled carbon nanotube–graphene hybrid nanostructures. J. Chem. Phys..

[9] Furtado C., Kopp R., Ni X., Sarrado C., Kalfon-Cohen E., Wardle B.L., Camanho P.P. (2024). J-Integral Experimental Reduction Reveals Fracture Toughness Improvements in Thin-Ply Carbon Fiber Laminates with Aligned Carbon Nanotube Interlaminar Reinforcement. ACS Appl. Mater. Interfaces.

[10] Nouioua A., Ben Salem D., Ouakouak A., Guergazi S., Abdelli A., Goma D., Gatica J.M., Vidal H. (2025). Ultra-Efficient Removal of Crystal Violet Dye Using Industrial Brine and Horn-Derived Biochar: Synergistic Action of Salting-Out/Adsorption. Toxics.

[11] Sharma S.K., Miladinović S., Sharma L.K., Gajević S., Sharma Y., Sharma M., Čukić S., Stojanović B. (2026). Graphene/CNT Nanocomposites: Processing, Properties, and Applications. Nanomaterials.

[12] Fedotov A.F. (2022). Mori-Tanaka experimental-analytical model for predicting engineering elastic moduli of composite materials. Compos. B Eng..

[13] Ganesan R., Palacios J., Abraham J., Thomas S., Kalarikkal N. (2022). Characterization of the Dynamic Response of CNT-Reinforced-Polymer-Composite (CNTRPC) Materials Based on a Multiscale Approach. Handbook of Carbon Nanotubes.

[14] Manoj A.P., Pradeep P.V., Shabana M.P., Anagha K.S., Ramakrisnan V. (2026). The functional role of different carbon/semiconducting oxide hybrid nanostructures for photoelectrochemical (PEC) water splitting. Sustain. Chem. Energy Mater..

[15] Qiu Y., Wang Z., Owens A.C., Kulaots I., Chen Y., Kane A.B., Hurt R.H. (2014). Antioxidant chemistry of graphene-based materials and its role in oxidation protection technology. Nanoscale.

[16] Mujib S.B., Rasheed M., Arunachalam S.R., Singh G. (2024). Hybrid HfC-SiCN matrix for improved oxidation resistance of carbon fiber–reinforced mini-composites. Int. J. Ceram. Eng. Sci..

[17] Yao C., Shen C. (2025). Kinetic analysis on low-temperature oxidation of wood pellets by isothermal microcalorimetry. Fire Mater..

[18] Zaccardi F., Toto E., Marra F., Santonicola M.G., Laurenzi S. (2023). Hybrid Carbon Nanocomposites Made of Aerospace-Grade Epoxy Showing Synergistic Effects in Electrical Properties and High Processability. Polymers.

[19] Yaqoob S., Ali Z., D’Amore A., Lo Schiavo A., Petraglia A., Rubino M. (2025). Enhanced Mechanical and Electrical Performance of Epoxy Nanocomposites Through Hybrid Reinforcement of Carbon Nanotubes and Graphene Nanoplatelets: A Synergistic Route to Balanced Strength, Stiffness, and Dispersion. J. Compos. Sci..

[20] Li W., Dichiara A., Bai J. (2013). Carbon nanotube–graphene nanoplatelet hybrids as high-performance multifunctional reinforcements in epoxy composites. Compos. Sci. Technol..

[21] Rajabifar N., Ghanemi S., Rostami A., Bahrami M. (2024). Synergistic impact of hybrid carbon nanotube and graphene on crystallinity and thermo-mechanical behavior of polymer blends. Polym. Compos..

[22] Liu B., Sun J., Zhao J., Yun X. (2025). Hybrid graphene and carbon nanotube–reinforced composites: Polymer, metal, and ceramic matrices. Adv. Compos. Hybrid Mater..

[23] Baniasadi H., Farzan A., McCord M.R.Y., Silva P.E.S., Abdi B., Paganelli Z., Vapaavuori J., Tehrani A., Niskanen J. (2025). Photocurable cellulose-based composites with PEGylated graphene oxide for leakage-free thermal energy storage and photothermal applications. Int. J. Biol. Macromol..

[24] Kudus M.H.A., Zakaria M.R., Omar M.F., Hafi Othman M.B., Akil H.M., Nabiałek M., Jeż B., Abdullah M.M.A.B. (2021). Nonisothermal Kinetic Degradation of Hybrid CNT/Alumina Epoxy Nanocomposites. Metals.

[25] Choudhury A., Bhowmick A.K., Ong C., Soddemann M. (2010). Effect of Various Nanofillers on Thermal Stability and Degradation Kinetics of Polymer Nanocomposites. J. Nanosci. Nanotechnol..

[26] Liu H., Ji X., Wang W., Zhou L. (2024). 3D-Networks Based Polymer Composites for Multifunctional Thermal Management and Electromagnetic Protection: A Mini Review. Materials.

[27] Yu C., Wu T., Yang F., Rao W., Zhao H.-B., Zhu Z. (2022). Construction of hetero-structured nanohybrid relying on reactive phosphazene towards flame retardation and mechanical enhancement of epoxy resins. Eur. Polym. J..

[28] Drozdov A. (2007). A model for thermal degradation of hybrid nanocomposites. Eur. Polym. J..

[29] Su S.P., Xu Y.H., China P.R., Wilkie A.A., McNally T., Pötschke P. (2011). Thermal degradation of polymer–carbon nanotube composites. Polymer–Carbon Nanotube Composites: Preparation, Properties and Applications.

[30] Fenta E.W., Mebratie B.A. (2024). Advancements in carbon nanotube-polymer composites: Enhancing properties and applications through advanced manufacturing techniques. Heliyon.

[31] Behdinan K., Moradi-Dastjerdi R., Safaei B., Qin Z., Chu F., Hui D. (2020). Graphene and CNT impact on heat transfer response of nanocomposite cylinders. Nanotechnol. Rev..

[32] Akinyi C., Iroh J.O. (2023). Thermal Decomposition and Stability of Hybrid Graphene–Clay/Polyimide Nanocomposites. Polymers.

[33] Ozawa T. (1965). A new method of analyzing thermogravimetric data. Bull. Chem. Soc. Jpn..

[34] Flynn J.H., Wall L.A. (1966). General treatment of the thermogravimetry of polymers. J. Res. Natl. Bur. Stand. Sect. A Phys. Chem..

[35] Akahira T., Sunose T. (1971). Method of determining activation deterioration constant of electrical insulating materials. Res. Rep. Chiba Inst. Technol. (Sci. Technol.).

[36] Friedman H.L. (1964). Kinetics of thermal degradation of char-forming plastics from thermogravimetry. Application to a phenolic plastic. J. Polym. Sci. Part C.

[37] Nobile M.R., Raimondo M., Naddeo C., Guadagno L. (2020). Rheological and Morphological Properties of Non-Covalently Functionalized Graphene-Based Structural Epoxy Resins with Intrinsic Electrical Conductivity and Thermal Stability. Nanomaterials.

[38] Guadagno L., Naddeo C., Sorrentino A., Raimondo M. (2023). Thermo-Mechanical Performance of Epoxy Hybrid System Based on Carbon Nanotubes and Graphene Nanoparticles. Nanomaterials.

[39] Vyazovkin S., Burnham A.K., Criado J.M., Pérez-Maqueda L.A., Popescu C., Sbirrazzuoli N. (2011). ICTAC Kinetics Committee recommendations for performing kinetic computations on thermal analysis data. Thermochim. Acta.

[40] Šimon P. (2005). Considerations on the single-step kinetics approximation. J. Therm. Anal. Calorim..

[41] Šimon P., Hynek D., Maliková M., Cibulková Z. (2008). Extrapolation of accelerated thermooxidative tests to lower temperatures applying non-Arrhenius temperature functions. J. Therm. Anal. Calorim..

[42] Gillen K.T., Bernstein R., Derzon D.K. (2005). Evidence of non-Arrhenius behaviour from laboratory aging and 24-year field aging of polychloroprene rubber materials. Polym. Degrad. Stab..

[43] Šimon P. (2005). Single-step kinetics approximation employing non-Arrhenius temperature functions. J. Therm. Anal. Calorim..

[44] Guadagno L., Raimondo M., Vittoria V., Vertuccio L., Naddeo C., Russo S., De Vivo B., Lamberti P., Spinelli G., Tucci V. (2014). Development of epoxy mixtures for application in aeronautics and aerospace. RSC Adv..

[45] Raimondo M., Russo S., Guadagno L., Longo P., Chirico S., Mariconda A., Bonnaud L., Murariu O., Dubois P. (2015). Effect of incorporation of POSS compounds and phosphorous hardeners on thermal and fire resistance of nanofilled aeronautic resins. RSC Adv..

[46] Raimondo M., Guadagno L., Speranza V., Bonnaud L., Dubois P., Lafdi K. (2018). Multifunctional graphene/POSS epoxy resin tailored for aircraft lightning strike protection. Compos. Part B Eng..

[47] Guadagno L., Sorrentino A., Longo R., Raimondo M. (2023). Multifunctional Properties of Polyhedral Oligomeric Silsesquioxanes (POSS)-Based Epoxy Nanocomposites. Polymers.

[48] Tuffi R., D’Abramo S., Cafiero L.M., Trinca E., Vecchio Ciprioti S. (2018). Thermal behavior and pyrolytic degradation kinetics of polymeric mixtures from waste packaging plastics. Express Polym. Lett..

[49] Ferdeghini C., Guazzelli L., Pomelli C.S., Ciccioli A., Brunetti B., Mezzetta A., Vecchio Ciprioti S. (2021). Synthesis, thermal behavior and kinetic study of N-morpholinium dicationic ionic liquids by thermogravimetry. J. Mol. Liq..

[50] Raimondo M., Donati G., Milano G., Guadagno L. (2022). Hybrid composites based on carbon nanotubes and graphene nanosheets outperforming their single-nanofiller counterparts. FlatChem.

[51] Guadagno L., Vertuccio L., Naddeo C., Raimondo M., Barra G., De Nicola F., Volponi R., Lamberti P., Spinelli G., Tucci V. (2019). Electrical Current Map and Bulk Conductivity of Carbon Fiber-Reinforced Nanocomposites. Polymers.

[52] Guadagno L., Calabrese E., Longo R., Aliberti F., Vertuccio L., Catauro M., Raimondo M. (2025). Morphological and Spectroscopic Characterization of Multifunctional Self-Healing Systems. Polymers.

[53] Li Z., Pan D., Han Z., Kumar D.J.P., Ren J., Hou H., El-Bahy Z.M., Mersal G.A.M., Xu B.B., Liu Y. (2023). Boron nitride whiskers and nano alumina synergistically enhancing the vertical thermal conductivity of epoxy-cellulose aerogel nanocomposites. Adv. Compos. Hybrid Mater..

