# Supplementary material for: Kinetic Study of the Oxidative Thermal Degradation of Polymer Composites Loaded with Hybrid Nanostructured Forms of Carbon: Correlation with Electrical and Morphological Properties
Source: Polymers (Basel). 2026 May 8;18(10):1150. doi: 10.3390/polym18101150 (PMC13210792; doi:10.3390/polym18101150)
Supplement: Supplementary file 1 [file polymers-18-01150-s001.zip › polymers-4249790-supplementary.pdf]

Supplementary materials

Kinetic study of the oxidative thermal degradation of polymer composites loaded with hybrid nanostructured forms of carbon. Correlation with electrical and morphological properties

Annalisa Paolone [1], Francesco Trequattrini [1,2], Marialuigia Raimondo [3,*], Liberata Guadagno [3] and Stefano Vecchio Ciprioti [4,*]

[1] Consiglio Nazionale delle Ricerche—Istituto dei Sistemi Complessi, U.O.S. La Sapienza, Piazzale A. Moro 5, 00185 Roma, Italy; annalisa.paolone@cnr.it (A.P.)

[2] Dipartimento di Fisica, Sapienza Università di Roma, Piazzale A. Moro 5, 00185 Roma, Italy; francesco.trequattrini@uniroma1.it (F.T.)

[3] Dipartimento di Ingegneria Industriale, Università di Salerno, Via Giovanni Paolo II, 132, 84084 Fisciano, Italy; lguadagno@unisa.it (L.G.)

[4] Dipartimento di Scienze di Base ed Applicate per l'Ingegneria, Sapienza Università di Roma, Palazzina RM017, Via del Castro Laurenziano 7, 00161 Roma, Italy;

* Correspondence: mraimondo@unisa.it (M.R.); stefano.vecchio@uniroma1.it (S.V.C.)

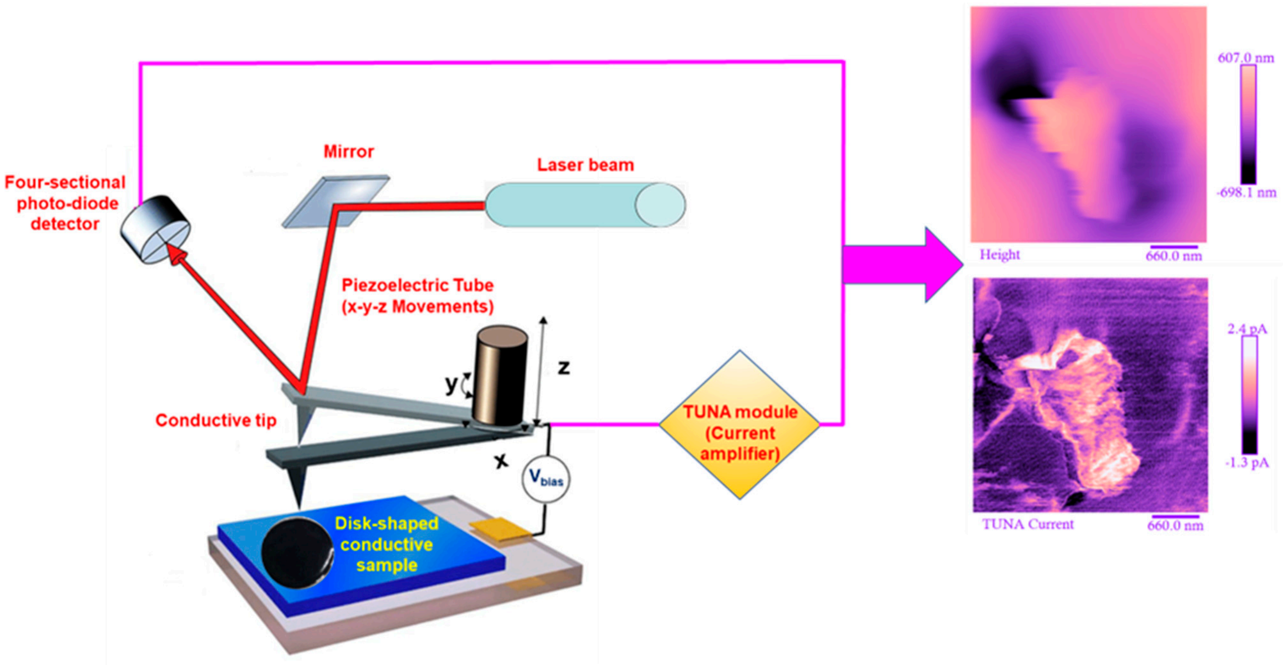

Figure S1. Experimental setup of TUNA instrument for acquiring topography and conductivity images simultaneously.

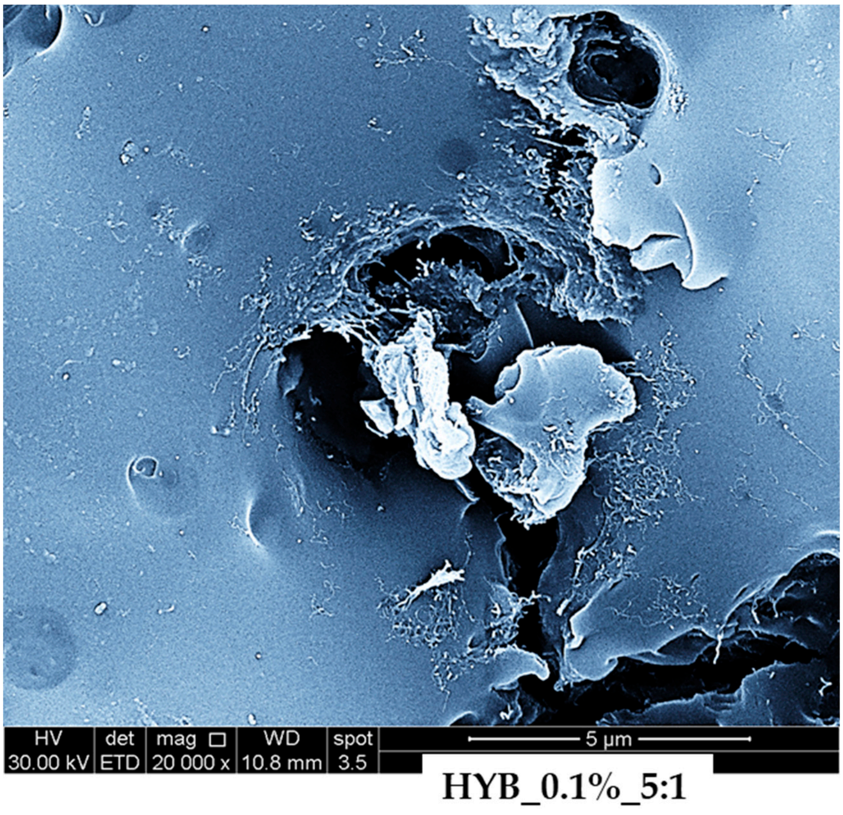

Figure S2. Field Emission Scanning Electron Microscopy (FESEM) image of the epoxy hybrid HYB_0.1% _5:1. (FESEM, mod. LEO 1525, Carl Zeiss SMT AG, Oberkochen, Germany).

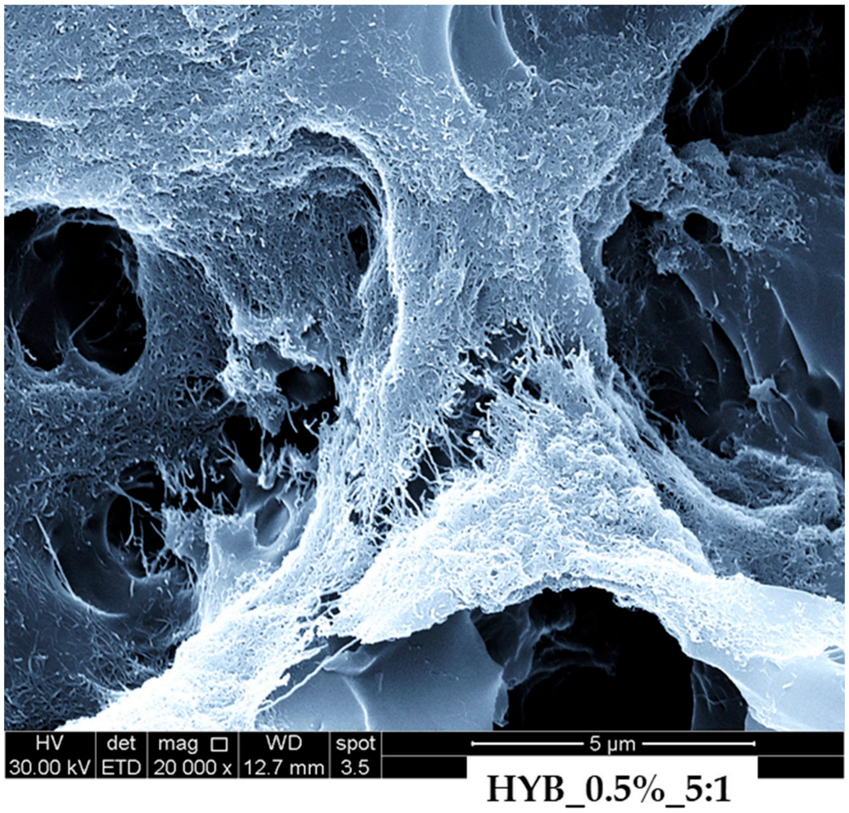

HYB_0.5%_5:1

Figure S3. Field Emission Scanning Electron Microscopy (FESEM) image of the epoxy hybrid HYB_0.5% _5:1. (FESEM, mod. LEO 1525, Carl Zeiss SMT AG, Oberkochen, Germany).
